# Indents

**Published:** 1916-10

**Authors:** 


### INDENTS

☞ **Brief Articles of Technical or Scientific Value will receive notice and comment. Contributions invited.**

*Ages of Carelessness.* Every one knows that few individuals between the ages of thirty and sixty take any constructive forethought for their physical welfare; few carry out any definite plans for regular daily exercise or proper breathing of fresh air. Fewer still have even a fair conception of their own physical make-up or their condition at any particular time; this fact is due likely both to lack of time and to reluctance to face the truth.—C. H. Forsyth, in *Journal A. M. A.*

*Advantage of the Card System.* If a card system is used, have the patient's card taken from its container, placed where the operator can refer to it before starting work for the patient. The operator will thus be enabled to proceed intelligently. The card should not be replaced in the container, until a charge has been made, for the operation. By using this method no charges will be overlooked.—Albert E. Converse, *Dental Review.*

*Flasking.* Before pouring the plaster of Paris into the flask to form the mold, thoroughly dust the inside of the flask with French chalk to prevent the plaster sticking to it. The vulcanized case can then be quite easily removed without bruising the flask.—*Ash's Journal.*

*Pyorrhea: A Preventable Disease.* When the dental profession realizes that pyorrhea is a preventable disease; that in its early stages it is easily prevented and cured; that only those cases are hopeless that are long neglected; that no drug or vaccine will ever of itself cure the disease, and that the dependence must be placed upon local treatment, they will have taken the first step toward the elimination from the mouths of their patients of the chief of mouth infections.—A. H. Merritt, D.D.S., *Dental Items of Interest.*

*Protecting Gum Margins in Partial Dentures.* The clinical experience of all offers a convincing proof of the first and most important essential in designing a partial denture or any kind of artificial restoration of lost teeth. The necessity for keeping all clasps, bands, half-bands, hoods, saddles, bars, etc., away from the free margin of the gum, and especially sinking them between the root and the free gum margin is imperative.—W. E. Cummer, D.D.S., *Oral Health.*

*Ionization in Sterilizing Root Canals.* As an agent in sterilizing root canals, there can be only one verdict—that ionization of zinc chloride is the best method at our disposal. A fine platinum or steel broach, with some cotton wool soaked in a five per cent. solution of $ZnCl_2$ and introduced into all canals before filling, will insure an absolutely aseptic canal filling. The zinc ions will penetrate the dentinal tubules, and any escaping through the apex will not irritate, but if anything, will stimulate and sterilize to perfection; in fact, the whole tooth structure could be saturated with zinc and insure no after trouble.—J. Polack, *Australian Dental Journal,* through *Dental Items of Interest.*

*One Method of Treating and Filling Root Canals.* The first step consists of the preparation and sterilization of the field of operation by the accepted method. The second step involves treatment and removal of the pulp by the accepted method. The third step, which may or may not be taken at the same time, is the dehydration of the canals in a manner identical with the dehydration of a histological specimen for microscopic examination. I use sixty, then eighty, then ninety-five per cent. grain alcohol, washing the canals thoroughly with each in subsequent baths, and drying by the accepted method. The fourth step consists of the introduction of a fluid mixture which is compatible with either unmodified dentin or with dentin which has been treated as described. This mixture consists of medicinally pure zinc oxide and guaiacol; and is of a consistency just too thick to flow or to drop from a small spatula. This substance is led into the canals in the usual way with a broach, attention first being given to the region of the apex, and then, a sufficient quantity of the material being present, by diminishing the length of thrust in an orderly way, the broach is gradually withdrawn and the canal remains filled. The excess of the material is then removed from the pulp chamber and in the fifth step the pulp chamber is filled with a good oxyphosphate cement. For this purpose I have used Weston's Cement for some time, with unvarying satisfaction. The sixth step deals with the completion of the operation by filling or other means.—Paul R. Manning, D.M.D., *Dental Cosmos.*

*Dr. Hillyer's Catechism.* I affirm the principle that teeth move in function.

That their mobility is essential to the maintenance of a physiologic equilibrium by virtue of inducing a constantly active circulation.

That different teeth sustain different stresses and move in different directions.

That teeth used as abutments for bridges must be allowed to retain their individual tooth motion.

That artificial restorations, known as bridges, must move to a limited extent with the underlying mucosa.

That these restorations must move in the same directions and to the same limited extent in which and to which the natural teeth moved when they were in proper place.

That parallel abutments and attachments are absolutely essential for correct bridge restorations.

That it is nothing short of criminal to destroy a pulp in a tooth not involved by decay in fairly normal position and distally to the cuspid.

That the destruction of such teeth is unnecesary in order to make them available for bridge abutments.

The uselessness of clasps, the folly of buttons, the unreliability of universal joints, the crudeness of split bars and the malicious influence all the foregoing have upon the human organism.

The correctness of applying geometric principles obtaining the necessary limited mobility in bucco-lingual and linguo-buccal directions.

I affirm the necessity of dissipating by the above means the stress upon a bridge before the elastic limit of the underlying tissue has been reached.—*Journal Allied Societies.*

*Apoplexy During Administration of Somnoform.*

Mary C., age sixty-one, attended the hospital for the purpose of having a number of carious teeth removed. She was extremely nervous, and it was agreed that she should have a general anesthetic. Somnoform was chosen, and given by the semi-open method. She made an uneventful recovery, but, on attempting to leave the chair, complained of her inability to walk without assistance. The first sign of trouble was noticed by the drooping of the left corner of the mouth, and then gradual spreading paralysis of the left side of the body. Two hours later the woman was completely paralyzed down the one side. The only history of illness she could relate was that she had been suffering from her back for some time. She is now an inmate of one of the general hospitals, and is making slow progress towards recovery. I can not find a similar case recorded. The anesthetic was probably an exciting cause, but I consider that the nervous excitement prior to the operation must have been the original factor in bringing about the cranial hemorrhage.—E. S. F., *Australian Journal of Dentistry.*

*Better Dentists.* "As we see things today, it means there will be far less surgery and less sickness when the full benefit of the knowledge of modern dentistry and the cause of many diseases becomes known throughout the world. There should be four times as many dentists as there are today, and yet there is not room for more until they have educated the public to the necessity of their work. No matter how far advanced surgeons or medical men, or men in any line of endeavor may be in a community, they know they could not have done their work unless the people of that community had been educated to the necessity for such work and the benefits they would derive from it. Therefore, it is up to you dentists to institute an educational propaganda such as has been going out from the American Medical Association. You all know of their work during the past few years. They have distributed men throughout the country who are educating the public as regards the necessity for early examination and treatment of cancer, in order that something may be done to obtain such cases for treatment during the curable stage of the disease. Educational measures of this kind should be undertaken in all branches of medicine which deal with public health."—Dr. Chas. H. Mayo.

*Even the Eggs May Be Infected.* According to a Census Bureau estimate the egg industry furnishes an average of 210 eggs per year or four eggs per week for each person in the United States. Their annual value at the point of production is over $300,000,000. These statistics explain the widespread interest in all facts pertaining to eggs either with respect to their production, marketing, or dietary uses. Why a product so carefully packed away in a well lined shell should become infected and undergo deterioration so easily has been a puzzle to many persons. It is not generally understood that a certain amount of bacterial infection may be found even in freshly laid eggs. Of more than 2,500 fresh eggs examined at the Rhode Island Experiment Station over eight per cent. showed bacterial infection in the yolk. None of the whites examined showed infection. The percentage of infection obtained for individual hens per year varied between two and eight-tenths and fifteen, the average being nine. No hen laid all sterile eggs during any full year. The percentage of infection for infertile and for fertilized eggs was essentially the same. The invading organisms occurring included forty bacterial types, among which were eleven cocci, twenty-eight rods, and one spirillum. The most probable source of primary egg infection is the ovaries of the fowl, which become infected by bacteria escaping through the intestinal wall into the portal circulation. The nature of the bacterial species occurring in the primary infection makes clear the fact that primary infection plays no role in bringing about the decomposition of eggs. For the factors determining this result we must look mainly to the secondary infections. The nature and extent of the normal primary infection stands in no causal relation to embryo mortality in incubating eggs, and losses in "dead-in-shell" eggs can not be explained on these grounds.

